# Isolation of a novel alkaline-stable lipase from a metagenomic library and its specific application for milkfat flavor production

**DOI:** 10.1186/1475-2859-13-1

**Published:** 2014-01-04

**Authors:** Qing Peng, Xu Wang, Meng Shang, Jinjin Huang, Guohua Guan, Ying Li, Bo Shi

**Affiliations:** 1 State Key Laboratories for Agro-biotechnology and College of Biological Sciences, China Agricultural University, Beijing 100193, P. R. China; 2 Feed Research Institute, Chinese Academy of Agricultural Sciences, Beijing 100081, P. R. China

**Keywords:** Metagenomic library, Alkaline-stable lipase, Myristic acid, Palmitic acid, Milkfat flavor

## Abstract

**Background:**

Lipolytic enzymes are commonly used to produce desired flavors in lipolyzed milkfat (LMF) manufacturing processes. However, the choice of enzyme is critical because it determines the final profile of fatty acids released and the consequent flavor of the product. We previously constructed a metagenomic library from marine sediments, to explore the novel enzymes which have unique properties useful in flavor-enhancing LMF.

**Results:**

A novel lipase Est_p6 was isolated from a metagenomic library and was expressed highly in *E.coli*. Bioinformatic analysis indicated that Est_p6 belongs to lipolytic enzyme family IV, the molecular weight of purified Est_p6 was estimated at 36 kDa by SDS-PAGE. The hydrolytic activity of the enzyme was stable under alkaline condition and the optimal temperature was 50°C. It had a high specific activity (2500 U/mg) toward pNP butyrate (pNP-C4), with *K*_m_ and *V*_max_ values of 1.148 mM and 3497 μmol∙min^-1^∙mg^-1^, respectively. The enzyme activity was enhanced by DTT and was not significantly inhibited by PMSF, EDTA or SDS. This enzyme also showed high hydrolysis specificity for myristate (C14) and palmitate (C16). It seems that Est_p6 has safety for commercial LMF flavor production and food manufacturing processes.

**Conclusions:**

The ocean is a vast and largely unexplored resource for enzymes. According the outstanding alkaline-stability of Est_p6 and it produced myristic acid and palmitic acid more efficiently than other free fatty acids in lipolyzed milkfat. This novel lipase may be used to impart a distinctive and desirable flavor and odor in milkfat flavor production.

## Background

Partial hydrolysis (lipolysis) of milkfat imparts special flavors to dairy products [1,2]. The modified milkfat displays distinct flavor notes depending on the degree of lipolysis, *e.g.*, a sensory note of acid-free richness at a very low degree of lipolysis, and buttery, creamy, or cheesy tastes at higher degrees [3]. Because of its effect on flavor, lipolyzed milkfat (LMF) is a widely used and important ingredient in the food industry [3].

Lipolytic enzymes are ubiquitous in nature and play essential roles in LMF manufacturing processes [4]. Based on their substrate preference, these enzymes are categorized as lipases (EC 3.1.1.3) that hydrolyze long-chain acylglycerols (carbon chain length >10) and esterases (EC 3.1.1.1) that hydrolyze short-chain acylglycerols (carbon chain length ≤10) [5]. Both lipases and esterases are commonly used to produce desired flavors in dairy products. The choice of enzyme is critical because it determines the final profile of fatty acids released and the consequent flavor of the product. Short-chain fatty acids (C4-C8) generally impart a cheesy taste. The concentration of long-chain fatty acids must be kept below a certain threshold to avoid a soapy taste [6,7]. The ratio of short-chain to long-chain fatty acids in their free forms is therefore an important parameter and is highly dependent on the enzyme(s) utilized.

A novel approach, “metagenomics”, that does not involve culturing of microorganisms, permits screening for novel lipolytic enzymes with industrial potential from a variety of environments [8-10]. Aquatic and marine microorganisms are poorly known in comparison with terrestrial microorganisms. Screening of lipolytic enzymes from marine environments has the potential to greatly expand the range of enzyme activities and specificities available to us.

We previously constructed a metagenomic library from marine sediments [11]. We now describe the identification and characterization of a novel lipolytic enzyme, Est_p6, which has unique properties useful in the manufacture of flavor-enhancing LMF.

## Results and discussion

### Selection of novel lipolytic enzyme Est_p6

One open reading frame (ORF) from our marine sediment metagenomic library revealed with plasmid pNLE6 (GenBank: GQ168545) was annotated as a putative lipolytic enzyme based on the function of its conserved domain and designated as Est_p6 (GenBank: ACZ16565). Est_p6 encoded 357 amino acids. The protein showing 83% identity with a cold-active esterase from another deep-sea metagenomic library (GenBank: ADA70028) [12] and 59% identity with an esterase from *Vibrio splendidus* LGP32 (GenBank: YP_002394831).

**Figure 1:**
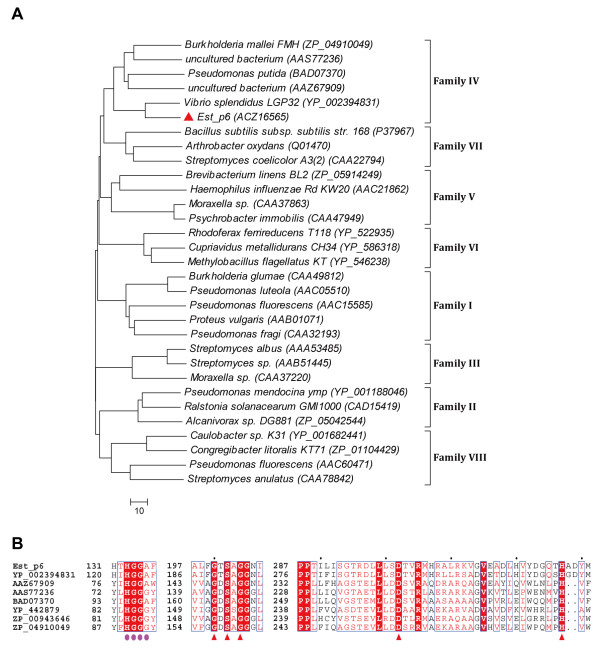
**Bioinformatic analysis of Est_p6. (A)** Unrooted neighbor-joining phylogenetic tree of Est_p6 (red triangle) and related bacterial lipolytic enzymes, based on conserved sequence motifs. The a.a. sequences of the other enzymes were obtained from published data. Sequence alignment was performed using ClustalW version 2.0, and the tree was created using MEGA version 5.2. Scale bar: number of a.a. substitutions per site. **(B)** Conserved sequence blocks from multiple sequence alignment between Est_p6 and family IV members. Sequence alignment was performed using ClustalW and ESPript. Conserved sequences are indicated by boxes, and similar sequences are indicated by colored background. The catalytic triads (red triangles) and the typical motif of family IV (pink circles) are identical.

Bioinformatic analysis indicated that Est_p6 belongs to lipolytic enzyme family IV (Figure 1A) and has striking amino acid sequence similarity to mammalian hormone-sensitive lipase (HSL) [5]. Extensive amino acid sequence analysis (Figure 1B) identified the catalytic triad of Est_p6 as Ser^202^-Asp^302^-His^327^ (Ser^202^ is contained within the classical GXSXG pentapeptide motif at amino acid positions 200–204) and a typical family IV HGGG(A)X motif composed of the H^133^GGAF sequence.

### Expression and purification of Est_p6

SignalP and TMHMM analysis revealed the presence of a predicted signal peptide in the initial 26 amino acids of Est_p6, suggesting that Est_p6 may be a transmembrane protein. The target gene was therefore amplified from the 27th amino acid (without the signal peptide sequence), cloned into pET28 vector with a 6 × His tag at the C-terminus, and transformed into *E. coli* BL21 (DE3) for expression.

**Figure 2:**
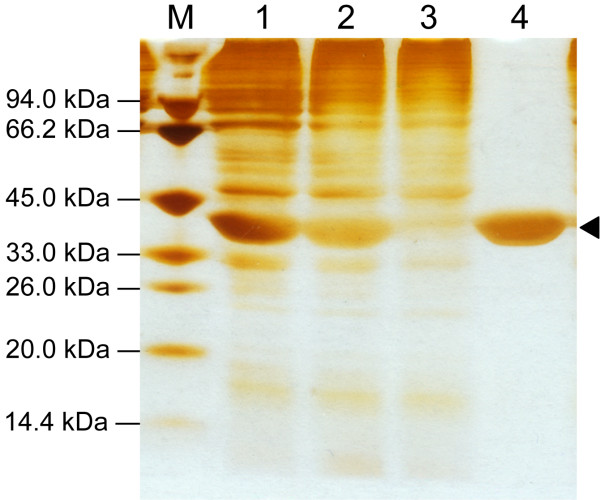
**Purification of recombinant Est_p6.** Proteins recovered during various purification steps as described in the text were separated by SDS-10% polyacrylamide gel electrophoresis and stained with Coomassie Brilliant Blue R-250. Lane M, molecular weight standards (kDa values indicated at left); Lane 1, total cell lysate; Lane 2, soluble fragment of cell lysate; Lane 3, flow-through fraction; Lane 4, 250 mM imidazole elution fraction. Purified Est_p6 was indicated by the triangle pointer.

The target protein Est_p6 was successfully purified by Ni-NTA-agarose chromatography and appeared as a single band on SDS-PAGE with molecular weight corresponding to the predicted value 36 kDa (Figure 2, line 4). The purified enzyme had a high specific activity (2500.5 U/mg) using pNP-C4 as a substrate and an overall purification yield of 87% (Table 1).

**Table 1:** Purification parameters of Est_p6

**Step**	**Volume (ml)**	**Activity (U/ml)**	**Protein (mg/ml)**	**Specific activity (U/mg)**	**Yield (%)**	**Purification (-fold)**
Soluble fragment	20.0	2907.1	6.14	473.5	100	1.0
Ni-NTA	17.0	2975.6	1.19	2500.5	87.0	5.0

### Characterization of Est_p6

#### Effect of pH and temperature on enzyme activity

**Figure 3:**
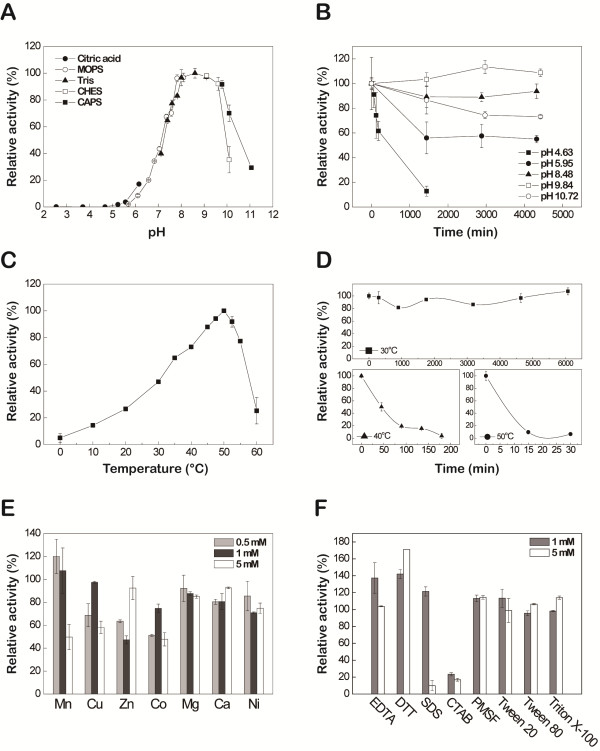
**Characterization of Est_p6. (A)** Effect of pH on Est_p6 activity, measured at 50°C for 3 min in 50 mM buffer. The buffers used were sodium citrate (●), MOPS (○), Tris–HCl (▲), CHES (□), and CAPS (■). **(B)** Effect of pH on stability of Est_p6, treated at 0°C for 0–4000 min in various buffers. Residual enzyme activity was measured at 50°C in 50 mM Tris–HCl buffer, pH 8.60. **(C)** Effect of temperature on Est_p6 activity, measured for 3 min in 50 mM Tris–HCl buffer, pH 8.60. **(D)** Effect of temperature on Est_p6 stability. Est_p6 was incubated in 50 mM Tris–HCl buffer, pH 8.60, at 30, 40, or 50°C for various durations, and residual activity was measured at 50°C for 3 min. **(E)** Effect on Est_p6 activity of various metal ions as indicated, at concentrations of 0.5, 1, and 5 mM. **(F)** Effect on Est_p6 activity of various detergents and inhibitors at concentrations of 1 and 5 mM. The compounds tested were non-ionic surfactants (Tween 20, Tween 80, Triton X-100), ionic surfactants (SDS, CTAB), inhibitors (EDTA, PMSF), and a reducing agent (DTT). The maximal activity was defined as 100% and the relative activity is shown as a percentage of maximal activity **(A-D)**, the 100% activity is shown as the activity that measured under standard conditions without metal cation or organic solvent **(E, F)**.

The optimal pH for Est_p6 activity was studied using pNP-C4 as a substrate and a pH range of 3–11. Est_p6 displayed >90% of its maximal activity within the pH range 8–10 and an optimal pH of ~8.60 (Figure 3A).

Est_p6 was highly resistant to alkaline inactivation (Figure 3B). After incubation for 3 days in the pH range 8–11, Est_p6 displayed >70% residual activity, with maximal stability at pH 9.84. Under acidic conditions, Est_p6 retained ~60% residual activity after 3 days at pH 5.95, but lost all activity after 1 day at pH 4.63. On the basis of these findings, Est_p6 was considered to be a highly alkaline-stable lipase.

The activity of Est_p6 increased steadily as temperature was increased from 0 to 50°C and then decreased sharply from 50 to 60°C, indicating an optimal temperature of 50°C (Figure 3C). For determination of thermostability, the purified enzyme was incubated in a temperature range of 30-50°C and the residual activity was measured. Est_p6 was highly stable at 30°C, with high activity maintained up to 4 days, whereas the half-life was only ~50 min at 40°C (Figure 3D).

#### Substrate specificity

The substrate specificity of purified Est_p6 was characterized using pNP esters (Table 2). The enzyme displayed hydrolytic ability for pNP esters in the C2 to C12 range. The highest specific activity was found for pNP butyrate (C4) (*V*_max_ = 3497 μmol∙min^-1^∙mg^-1^), whereas activities for long-chain pNP esters of myristate (C14) and stearate (C18) were low. In view of these findings, Est_p6 is classified as a lipase. Estimation of catalytic efficiency based on the *k*_cat_/*K*_m_ value indicated that pNP butyrate (C4) was the best substrate for Est_p6 among the pNP esters tested.

**Table 2:** Kinetic parameters of Est_p6 for various pNP esters

**Substrate (pNP ester)**	***K***_**m**_**(mM)**	***V***_**max**_**(μmol∙min**^**-1**^**∙mg**^**-1**^**)**	***k***_**cat**_**(s**^**-1**^**)**	***k***_**cat**_**/*K***_**m**_**(s**^**-1**^ **mM**^**-1**^**)**
Acetate (C2)	2.644	212.695	133.303	50.413
Butyrate (C4)	1.148	3496.626	2191.452	1908.445
Caprylate (C8)	0.322	914.645	573.239	1779.330
Caprate (C10)	1.280	163.068	102.200	79.815
Laurate (C12)	0.149	0.795	0.499	3.339

Lipases are distinguished from esterases on the basis of substrate preferences. Lipase activity is directed to substrates with acyl chain lengths of >10 carbon atoms that are generally reactive in the oil–water interface [13,14]. Several previous reports have described the cloning and characterization of metagenomic lipolytic enzymes from family IV, which are generally classified as esterases on the basis of substrate preference [15-17]. In the present study, Est_p6 was found to hydrolyze pNP-esters up to pNP-laurate (C12) and milkfat esters up to palmitate (C16), and was therefore classified as a lipase (not an esterase) from family IV. The overall substrate specificities of esterases and lipases have been attributed to numerous features, including differences in the size and overall hydrophobicity or hydrophilicity of the substrate-binding pocket [18]. In regard to these properties, the substrate-binding sites of Est_p6 are probably different from those of family IV esterases.

#### Effects of metals, detergents, and inhibitors on enzyme activity

The effect on Est_p6 activity of various metal ions (Mn^2+^, Cu^2+^, Zn^2+^, Co^2+^, Mg^2+^, Ca^2+^, Ni^2+^) was tested at concentrations of 0.5, 1.0, and 5.0 mM. None of these tests showed any significant stimulation or inhibition of enzyme activity (Figure 3E). EDTA had no inhibitory effect on enzyme activity but had a slight stimulatory effect at low concentration (Figure 3F). DTT, a strong reducing agent, had a clear dose-dependent stimulatory effect on enzyme activity. The ionic detergents tested (such as SDS, CTAB) had a strong inhibitory effect on enzyme activity, whereas activity was not significantly affected by nonionic detergents (Tween 20, Tween 80, Triton X-100) and by PMSF.

Lipolytic enzymes belong to the class of serine hydrolases, and in most cases their activity is irreversibly inhibited by PMSF. In the present study, PMSF had no effect on Est_p6 activity. This is an unusual finding in lipolytic enzymes [11,19,20]. The inhibitory effect of PMSF may be eliminated by a lid structure in lipases [21]. The predicted tertiary structure of Est_p6 did not show strong identity with any known structure in the Protein Data Bank (PDB; URL: http://www.rcsb.org/pdb/home/home.do), suggesting that the structure is probably novel and still require modeling for further analysis of its role.

Est_p6 displayed high catalytic activity and stability under alkaline pH values up to 9.84 and a rapid loss of activity at pH values <7, indicating that Est_p6 is an alkaline-active and -stable lipase. Tests of two ionic surfactants, SDS and CTAB, showed that SDS had a stronger inhibitory effect on Est_p6 lipolytic activity. SDS is an anionic surfactant, and CTAB is a cationic surfactant. Thus, Est_p6 is evidently more tolerant of alkaline reaction systems and may therefore have useful application in a variety of cleaning products.

The catalytic activity of Est_p6 was enhanced by DTT. DTT is commonly used to reduce disulfide bonds of proteins and, more generally, to prevent the formation of intramolecular and intermolecular disulfide bonds between cysteine residues of proteins. According to the SignalP and HMMTOP prediction, Est_p6 is a membrane protein. The removal of its signal peptide for heterologous expression may have resulted in differing degrees of intermolecular assembly. The addition of DTT reduces such assembly and exposes the enzyme active site, better reflecting increases in enzyme activity.

### Potential application of Est_p6 in LMF preparation

**Figure 4:**
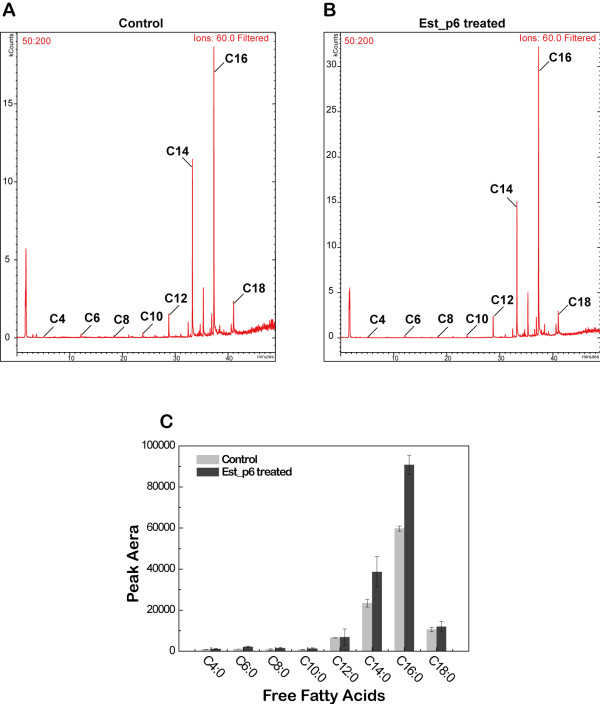
**GC-MS chromatograms showing changes in free fatty acid composition of butter milkfat resulting from Est_p6 treatment. A** and **B**: ordinate = peak height; abscissa = reaction time. **(A)** Untreated butter milkfat (control). **(B)** Est_p6-treated milkfat. **(C)** Comparison of peak areas of untreated vs. treated milkfat. The chromatograms were obtained by an ions filter with a typical fragment of fatty acids (-CH_2_COOH) approximate at 60 ± 1. The peak areas for myristic acid and palmitic acid were increased significantly by Est_p6 treatment.

GC-MS analysis was used to assess the ability of Est_p6 to hydrolyze butter milkfat. The control (Figure 4A) shows the volatile fatty acids with even numbers of carbon atoms that occur naturally in butter. The peak area of each volatile fatty acid was clearly increased by Est_p6 treatment (Figure 4B). In particular, the areas of myristic acid (C14) and palmitic acid (C16) increased by 65.5% and 52.0%, respectively (Figure 4C). Thus, Est_p6 treatment of butter milkfat could produce a richer flavor of volatile fatty acids, particularly the distinctive flavors of myristic and palmitic acid.

Analysis of volatile fatty acid composition (Table 3) showed that the flavor notes of butter were not greatly changed by Est_p6 treatment. In comparison with corresponding values from untreated butter, the percentages from Est_p6-treated butter were very similar for short-chain (C4-C8) fatty acids, slightly higher for myristic (C14) and palmitic (C16) acids, and slightly lower for lauric (C12) and stearic (C18) acids. Thus, Est_p6 treatment of butter maintains the cheesy flavor produced by short-chain fatty acids and enhances the special flavor produced by myristic and palmitic acids.

**Table 3:** Changes in fatty acid content in Est_p6-treated butter compared with untreated butter

**Fatty acid**	**Untreated butter (%)**	**Est_p6-treated butter (%)**	**Change (%)**
Butyric acid (C4:0)	0.761	0.726	-0.035
Caproic acid (C6:0)	1.051	1.436	+0.384
Octanoic acid (C8:0)	0.822	1.019	+0.197
Capric acid (C10:0)	0.696	0.881	+0.185
Lauric acid (C12:0)	6.305	4.463	-1.842
Myristic acid (C14:0)	22.542	25.023	+2.480
Palmitic acid (C16:0)	57.592	58.709	+1.117
Stearic acid (C18:0)	10.231	7.745	-2.486

The hydrolytic activity of Est_p6 was directed to pNP-esters with short to medium chain length (C2-C12). The highest *k*_cat_/*K*_m_ value (1908 s^-1^. mM^-1^) was found for pNP-C4, indicating that this was the most appropriate substrate for kinetic assay. The lowest *K*_m_ value (0.15 mM) was found for C12, suggesting that the pNP-C12 structure is closer to the natural substrate of Est_p6. This idea was supported by results of milkfat hydrolysis assays, which showed highest specific activities for myristate (C14) and palmitate (C16).

Controlled hydrolysis of milkfat by lipases has been applied in the food industry since the early 1990s, to produce flavor compounds used in bakery products (bread, cakes, cookie mixes), cereal products (flakes), candies (chocolate products, toffees), dairy products (coffee whiteners, confectionary creams, cheese, butter spreads), and a variety of other products including popcorn seasoning, sauces, salad dressings, and snack foods [22,23]. Several commercial lipases with differing LMF flavor profiles have been developed and utilized in current LMF flavor production and food manufacturing processes. These commercial lipases, which include Palatase 20000 L, MER, AY30G, Snow plum blossom, and Calf PGE, are commonly used for milkfat hydrolysis. They all have high specificity for short-chain fatty acids and produce primarily sharp, cheesy, or buttery odors [24]. Most previous studies have focused on specific release of short-chain fatty acids, which generally produce a strong cheesy flavor. Much less attention has been paid to volatile components containing medium-chain or long-chain fatty acids.

Myristic acid and palmitic acid are food additives that the FDA has approved for direct addition to foods. The volatile flavor of myristic acid and palmitic acid at low concentrations has been characterized as a blending of nectarine skin, mutton, and beef, and can impart a unique flavor to dairy products [25,26]. Est_p6 has high applicability and safety for commercial LMF flavor production and food manufacturing processes.

## Conclusion

The ocean is a vast and largely unexplored resource for enzymes and other organic compounds useful in the food industries. The gene encoding a novel lipase (Est_p6) was isolated from a metagenomic library constructed from marine sediments, and was expressed in *E.coli*. Est_p6 belongs to family IV of the lipolytic enzymes. It is stable under alkaline conditions and has a high specificity for myristate and palmitate. Est_p6 thus produces myristic acid and palmitic acid more efficiently than other free fatty acids in LMF, and imparts a distinctive and desirable flavor and odor in milkfat flavor production.

## Materials and methods

### Screening of lipolytic clones from a metagenomic library

Marine sediment samples obtained from the South China Sea were used to construct a small-insert metagenomic library as described previously [11]. Lipolytic clones were detected based on their ability to hydrolyze tributyrin (1%) substrate and to produce a clear halo around a library colony after 48 hr incubation at 37°C [27]. All lipolytic clones obtained were streaked to obtain single colonies, which were then re-tested for the ability to hydrolyze tributyrin. Positive clones were confirmed by plasmid isolation, restriction enzyme digestion, and sequencing.

### Bioinformatic analysis

Sequences were screened for vector contamination and subjected to quality trimming. Sequence assembly and analysis were performed using the DNAMAN program (version 6.0, Lynnon Corp., Pointe-Claire, Quebec, Canada). Open reading frames (ORFs) in each assembled sequence were identified using the ORF Finder program from the National Centre for Biotechnology Information (NCBI, Bethesda, Maryland, USA). The amino acid sequences of each ORF were used to find the best match and conserved domains with the NCBI’s protein-protein BLAST program. Signal peptides and transmembrane domains were predicted using server SignalP (URL: http://www.cbs.dtu.dk/services/SignalP/) and HMMTOP (URL: http://www.enzim.hu/hmmtop/index.php). Multiple sequence alignments were calculated using ClustalW (URL: http://www.ch.embnet.org/software/ClustalW.html) and exported by ESPript (URL:http://espript.ibcp.fr/ESPript/ESPript/). Phylogenetic relationships among lipolytic members in each protein family were analyzed by MEGA program (version 5.0, The Biodesign Institute, Tempe, Arizona, USA).

### Expression and purification of Est_p6

The Est_p6 gene was amplified from the positive plasmid pNLE6. The forward primer (5′- CATG**CCATGG**AGGAACTCCCTCCGATATT-3′) with the restriction enzyme site *Nco*I, and the reverse primer (5′- CCC**AAGCTT**CTCGAGGTGC TTGTCAAAG -3′) with *Hin*dIII, were designed to generate a C-terminal His-tag of the recombinant target protein. The *est_p6* gene was cloned into expressing vector pET-28a(+) and then transformed into *E. coli* BL21(DE3) cells. Transformants were grown on LB medium containing 50 μg ml^-1^ kanamycin at 37°C. When cells reached a certain density (OD_600_ = 0.5), they were induced for 12 hr with 0.5 mM isopropyl β-D-1-thiogalactopyranoside (IPTG) at 21°C. The target protein was eluted at imidazole concentration 250 mM by Ni-NTA (Qiagen, Germantown, Maryland, USA) affinity chromatography, and protein concentration was determined by the Lowry protein assay method [28]. The molecular weight of the purified protein was determined by SDS-PAGE.

### Enzyme characterization

#### Enzymatic activity

Lipase/esterase activity was determined by a spectrophotometric method using p-nitrophenyl (pNP) esters. The catalytic activity of Est_p6 was evaluated using pNP butyrate as a standard substrate for 3 min at 50°C. The assay mixture contained 1 mM pNP esters, 50 mM Tri-HCl buffer (pH 8.60), and 4% ethanol in a total volume of 1 ml. Absorbance was measured at 405 nm. One unit esterase activity was defined as the amount of enzyme required to release 1 μmol pNP in 1 min.

#### Optimal values and stability of pH and temperature

The optimal pH of purified Est_p6 was determined under standard conditions. The buffers used were 50 mM sodium citrate (pH 2.55-6.16), MOPS (pH 5.69-8.10), Tris–HCl (pH 7.12-9.10), CHES (pH 9.11-10.09), and CAPS (pH 9.78-11.06). pH stability was determined by incubating the assays at pH values ranging from 4.63 to 10.72 for ~3 days and measuring the residual activity. The optimal temperature was determined under standard conditions in the range 0-60°C. Temperature stability was determined by incubating the assays at temperatures ranging from 30 to 50°C for 30 min to 100 hr and measuring the residual activity.

#### Substrate specific activity

Substrate range and specific activity were determined under standard conditions using pNP esters with acyl chains of various lengths: pNP acetate (C2), pNP butyrate (C4), pNP caprylate (C8), pNP caprate (C10), pNP laurate (C12), pNP palmitate (C16), and pNP stearate (C18). Initial reaction velocities measured at various substrate concentrations were fitted to the Lineweaver-Burk transformation of the Michaelis-Menten equation. Kinetic analyses by curve fitting were performed with the Fit linear program (OriginLab Corp., Northampton, Massachusetts, USA).

#### Stability in divalent metal cation and organic solvents

The activity of purified Est_p6 was assayed under standard conditions in the presence of various potentially inhibitory reagents: divalent metal cations (Mn^2+^, Cu^2+^, Zn^2+^, Co^2+^, Mg^2+^, Ca^2+^, Ni^2+^) (0.5, 1.0, or 5.0 mM), the chelating agent ethylenediaminetetraacetic acid (EDTA), the antioxidant dithiothreitol (DTT), the inhibitor phenylmethylsulfonyl fluoride (PMSF), and detergents such as sodium dodecyl sulfate (SDS: 1.0 or 5.0 mM) and cetyltrimethylammonium bromide (CTAB: 1.0 or 5.0 mM). Stability in surfactants was determined by measuring the residual activity after incubation with 1% or 5% (v/v) Tween 20, Tween 80, or TritonX-100 for 1 hr at 30°C.

### Hydrolysis of milkfat lipids

The lipolysis of milkfat was performed under optimal reaction conditions. Commercial butter (5 g) and 5 ml Tris–HCl buffer (50 mM, pH 8.6) were reacted with Est_p6 (6250 U activity for pNP-C4) in a 125-ml Erlenmeyer flask shaken at 200 rpm in an incubator for 4 hr at 40°C. The mixture was placed in an 85°C water bath for 15 min immediately after the end of incubation to stop the hydrolysis reaction.

### Gas chromatography–mass spectrometry (GC-MS) analysis

The hydrolyzed product (1 g) and 2 ml ethanol were placed in a 10 ml airtight sample vial and kept in an incubator at 65°C for 1 hr. A portion of the headspace gas (volume ~1 ml) was withdrawn with a 2 ml syringe for GC-MS analysis.

GC-MS was performed using a Varian 240-MS in combination with a Varian 450-GC (Agilent, Santa Clara, California, USA). Compounds were separated on a capillary column (model DB-5MS, internal diameter 30 m × 0.25 mm, film thickness 0.25 μm; Agilent). The GC program was: oven temperature 45°C for 5 min, increase at 5°C/min to 250°C, 250°C for 3 min; carrier gas = helium with constant flow rate 1.5 ml/min; injection (1 ml) in split mode (1:20 split ratio); GC-MS transfer line temperature 200°C. MS operation was in full scan mode with range 50–500 amu. Compounds were identified by comparison of mass spectra with commercial mass spectral databases from the Main lib, Wiley, and NIST libraries.

## Abbreviations

CAPS: N-cyclohexyl-3-aminopropanesulfonic acid; CTAB: Cetyltrimethylammonium bromide; CHES: N-cyclohexyl-2-aminoethanesulfonic acid; MOPS: 3-(N-morpholino) propanesulfonicaci; SDS: Sodium dodecyl sulfate; EDTA: Ethylenediaminetetraacetic acid; DTT: DL-Dithiothreitol; PMSF: Phenylmethanesulfonyl fluoride.

## Competing interests

The authors declare that they have no competing interests.

## Authors’ contributions

YL, BS, QP initiated and coordinated the project. QP, WX and MS performed gene cloning and expression. QP, JJH and GHG were responsible for enzyme characterization. YL, BS provided critical discussion. QP and YL wrote the paper and all authors approved the final version of the manuscript.
